# Unique technique of surgery in an unusual variety of Scimitar syndrome: A Case Report

**DOI:** 10.1186/1749-8090-5-15

**Published:** 2010-03-25

**Authors:** Julia Nuebel, Katarzyna Januszewska, Markus Loeff, Daniel Theisen, Edward Malec, Robert Dalla-Pozza

**Affiliations:** 1 Pediatric Cardiology and Intensive Care, Ludwig-Maximilians-University, Munich, Germany; 2 Cardiac Surgery, Ludwig-Maximilians-University, Munich, Germany; 3 Department of Radiology, Ludwig-Maximilians-University, Munich, Germany

## Abstract

Scimitar syndrome is a rare congenital anomaly characterized by total or partial anomalous pulmonary venous drainage of the right lung to the inferior vena cava. We present a seven year old girl with a systolic murmur who was diagnosed as having a Scimitar syndrome with unusual drainage of the right pulmonary veins. The unique technique of surgery in this patient was appropriate to the unusual, previously not described anatomy.

## Background

Scimitar syndrome is a rare congenital anomaly characterized by total or partial anomalous pulmonary venous drainage of the right lung to the inferior vena cava causing a left-to-right shunt [1-5]. The descending pulmonary vein is visible as a curviliniear density along the right heart, reminding a Turkish sword on the chest radiogram. Associated anomalies are hypoplastic right pulmonary artery and hypoplastic right lung, abnormal bronchial anatomy (bronchopulmonary sequestrations) and systemic arterial supply to the right lung from the abdominal aorta. Occasionally, atrial septal defect, ventricular septal defect, coarctation of the aorta and dextrocardia are present [1,4,6]. Furthermore there is a female preponderance [2].

Despite the varying spectrum of this syndrome, about half of the patients are asymptomatic or mildly symptomatic at the time of diagnosis [7]. Since the syndrome may be undetected in asymptomatic patients, the true incidence is difficult to determine [2,3]. Two different types of Scimitar syndrome can be identified. The infantile form of scimitar syndrome resembles a rapidly proceeding form of congestive heart failure due to substantial right ventricular volume overload and has to be corrected early in life. Baffle repair of the anomalous vein is possible in this group but long-term complications are not encouraging. The adult form is usually detected after the first year of life and patients are often mildly symptomatic with a good outcome after intracardiac repair [4]. The first reported case of Scimitar syndrome was published in 1836 by Cooper [8] and the first reported successful physiological repair of the syndrome by Kirklin, Ellis and Wood in 1956 [9,10].

We present a seven year old girl with a systolic murmur who was diagnosed as having a Scimitar syndrome with unusual drainage of the right pulmonary veins.

## Case presentation

**Figure 1:**
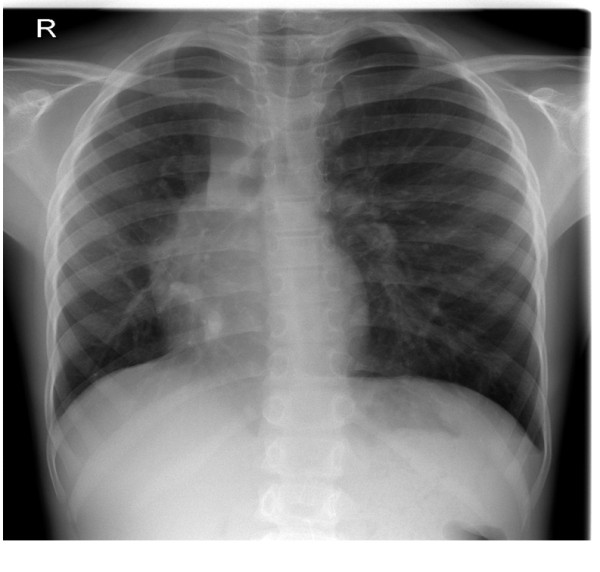
**Preoperative Chest X-ray showed a dextroposition and mesocardia with scimitar vein**.

A seven year old girl was evaluated for systolic heart murmur. Her examination was entirely normal except for the known murmur and right sided decreased lung sounds. The chest radiogram demonstrated hypoplasia of the right lung and shift of the mediastinal structures to the right (Figure 1).

Echocardiography showed mesocardia, dilated right ventricle and subdiaphragmal vein connected to the inferior vena cava to right atrium junction. A moderate tricuspid regurgitation was also noted without evidence for pulmonary hypertension.

To confirm the suspected diagnosis of Scimitar syndrome, we performed a MRI of the thorax which showed dextroposition and mesocardia as well as middle and lower right pulmonary veins connecting to the inferior vena cava. The right upper pulmonary veins were seen to drain into the superior vena cava in the region of the azygos vein. The pulmonary arteries were not hypoplastic, however the size of the right pulmonary artery (12 mm) was smaller than the left pulmonary artery (14 mm).

**Figure 2:**
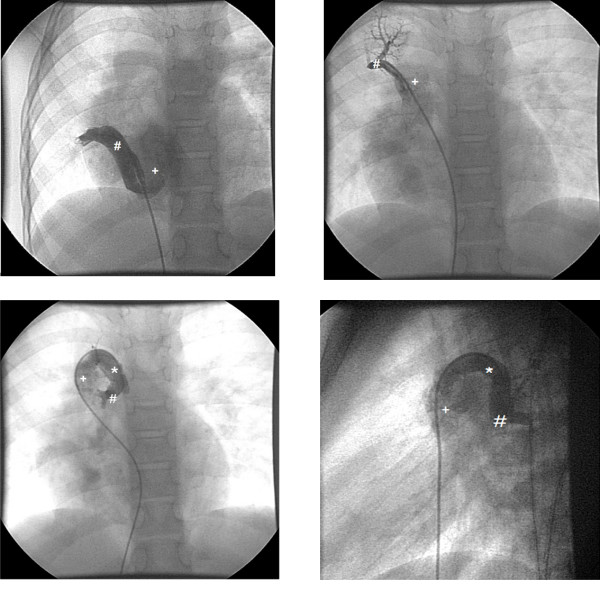
**Preoperative Angiography**. 1.a) Angiography in the Scimitar vein (#) and the connection to the inferior vena cava (+). 1.b) Angiography into an upper pulmonary vein (#) draining directly into the superior vena cava (+). 1.c) Angiography into a middle pulmonary vein (#) draining into the azygos vein (*) and then into the superior vena cava (+). 1.d) Lateral view: Angiography into a middle pulmonary vein (#) draining into the azygos vein (*) and then into the superior vena cava (+).

Cardiac catheterization was performed preoperatively to clarify the anatomy for exact planning of the operative strategy (Figure 2). The angiography demonstrated an anomalous drainage from the right lower lobe to the inferior vena cava (as shown in MRI), from the right upper lobe to the superior vena cava and middle pulmonary vein connected to the azygos vein. There were no systemic-to-pulmonary collateral arteries and an overall left-to-right shunt of 40% with normal pulmonary artery pressure.

According to the clinical, radiologic and hemodynamic findings, surgery was recommended at that time.

## Operative Technique

**Figure 3:**
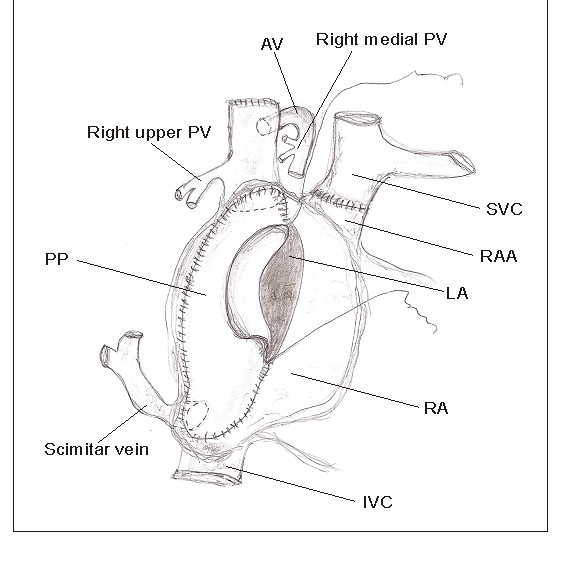
**Surgical technique**. AV - azygos vein, IVC - inferior vena cava, LA - left atrium, PP - pericardial patch, PV - pulmonary vein, RA - right atrium, RAA - right atrial appendage, SVC - superior vena cava.

Median sternotomy followed by aortic and bicaval cannulation was performed (innominate vein and left side of the inferior vena cava were cannulated). The patient was cooled with cardiopulmonary bypass to 18°C rectal temperature. During the cooling superior vena cava was transsected above the level of azygos vein and upper pulmonary vein drainage. The proximal end was oversown. After aorta cross-clamping, cardioplegic solution was administered and right atrium was opened. Atrial septal defect was enlarged by an extended resection of the septum primum. A large autologous pericardial patch was sown into right atrium to direct the flow from the azygos vein and upper pulmonary vein (through the opening of the superior vena cava) as well as the scimitar vein, through the atrial septal defect to the left atrium. The suture line around the scimitar vein was done in deep hypothermic circulatory arrest after removing of the venous cannula. During the rewarming, the anastomosis between distal part of superior vena cava and right atrial appendage was performed. The vena azygos was clipped distal to the connection with the middle right pulmonary vein (Figure 3).

## Postoperative Management

**Figure 4:**
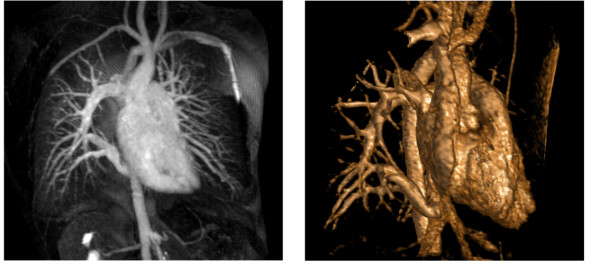
**Pre- and postoperative MRI**. 4.a) Preoperative MRI showed dextroposition and mesocardia, lower right pulmonary vein connecting to the inferior vena cava. Right upper and middle pulmonary veins draining in the superior vena cava and the azygos vein. 4.b) Postoperative MRI revealed the superior vena cava draining into the right atrium. The upper and middle pulmonary veins as well as the scimitar vein are redirected with a baffle into the left atrium.

The patient was extubated without any difficulty at the day of surgery. Due to pericardial effusion, we placed a pericardial drainage for 2 days. An early mobilisation was performed and anticoagulation with warfarin was started for a period of 3 months. The postoperative echocardiography showed a good function without any evidence of obstruction of the atrial baffle. We performed a postoperative MRI which revealed the superior vena cava draining into the right atrium. The upper and middle pulmonary veins as well as the scimitar vein were redirected with a baffle into the left atrium (Figure 4).

## Discussion

The etiology of Scimitar syndrome is unclear [11] but the defining characteristic anatomic feature is the partial anomalous pulmonary venous return [1]. Usually, there is a single vein that runs from the middle of the right lung to the cardiophrenic angle [3]. Another established variety is a doubled-arched vein in the upper and lower lung with drainage into the inferior vena cava [12]. Common associated anomalies are hypoplastic right pulmonary artery and lung, abnormal bronchial anatomy and systemic arterial supply to right lung from the abdominal aorta. Scimitar syndrome has a variable presentation such as severe respiratory insufficiency, cardiac failure [13], pulmonary hypertension, recurrent respiratory infections and heart murmur [6].

Our patient presented with heart murmur and was diagnosed at the age of seven years, so this case would be classified to the patients group of "adult" Scimitar syndrome [3,4]. In this patient we found an unknown variety with drainage of the right lower lobe to the inferior vena cava, from the right upper lobe to the superior vena cava and to the azygos vein and additionally an ASD. Since the right pulmonary artery was smaller than the left pulmonary artery, the pulmonary arteries were not hypoplastic.

Previously not described anatomy entailed a unique technique of surgery.

To confirm the suggested diagnosis and identify the specific course of the anomalous venous drainage, we performed echocardiography, chest radiogram, MRI and cardiac catheterization. According to the clinical and radiologic findings, surgery was recommended at that time. In general, surgical approaches are quite variable and controversial, due to anatomic and pathologic features presented in each case [14]. The classic operation encompasses construction of a long intra-atrial baffle from the entry point of the scimitar vein into the inferior vena cava to the left atrium through an ASD [5]. In our patient atrial septal defect was enlarged and autologous pericardial patch was sown into right atrium to direct the flow from the azygos vein, the upper pulmonary vein as well as the scimitar vein through the atrial septal defect to the left atrium.

After surgical repair, there was no clinical sign of cardiac failure. The postoperative course continued without any complications and the girl left hospital in a very good condition.

## Conclusion

Considering complex and unusual forms is required in patients with Scimitar syndrome to adapt the surgical treatment to the various types of anatomy. In our case, cardiac catheterization with angiography appeared to be the most appropriate diagnostic to confirm the anatomy. Actually, in this unusual variety of Scimitar syndrome surgery was successful and feasible.

## Competing interests

There is no founding or financial affiliation of any of the above named authors influencing the content of the manuscript or leading to a conflict of interest.

## Authors' contributions

All authors read and approved the final manuscript.

## Consent

Written informed consent was obtained from the patients parents for publication of this case report and any accompanying images. A copy of the written consent is available for review by the Editor-in-Chief of this journal.
